# Association between packed red blood cell transfusion and clinical deterioration in neonatal necrotizing enterocolitis: a systematic review and meta-analysis

**DOI:** 10.1080/07853890.2026.2675123

**Published:** 2026-05-24

**Authors:** Jinglan Huang, Qian Gao, Ziqing Xu, Hua Wang

**Affiliations:** a Department of Pediatrics, West China Second University Hospital, Sichuan University, Chengdu, China; b Key Laboratory of Birth Defects and Related Diseases of Women and Children of the Ministry of Education, Sichuan University, Chengdu, Sichuan, China

**Keywords:** Neonates, necrotizing enterocolitis, anemia, blood transfusion, clinical deterioration

## Abstract

**Background:**

No systematic review has evaluated the existing evidence regarding the association between packed red blood cell (pRBC) transfusion and clinical worsening of necrotizing enterocolitis (NEC) in neonates. This systematic review and meta-analysis was conducted to address this knowledge gap.

**Materials and methods:**

We searched the Cochrane Library, EBSCO, Embase, Web of Science, Google Scholar, and PubMed for studies on pRBC transfusion and NEC published before May 10, 2025. Relevant articles were selected through title, abstract, and full-text screening. English-language case-control studies or cohort studies, or randomized controlled trials involving newborns with NEC that compared pRBC transfusion with no transfusion and reported changes in NEC clinical status were included. Review articles, systematic reviews, case reports, editorials, animal studies, duplicate publications, and studies with incomplete data were excluded.

**Results:**

Five studies involving 971 neonates with NEC were included. The pooled analysis demonstrated a potential association between pRBC transfusion and clinical deterioration of NEC in neonates (odds ratio: 6.05, 95% confidence interval: 3.02–12.14).

**Conclusions:**

pRBC transfusion was associated with an exacerbation of NEC in neonates. However, these findings should be interpreted cautiously because of the small number of eligible studies included in this meta-analysis, and future large-scale, well-designed studies are needed to confirm the observed association.

## KEY MESSAGES

1. Published studies have identified an association between pRBC transfusion and NEC progression; however, whether this association is causal remains unclear.
2. Despite consistent evidence from observational studies suggesting a potential association between pRBC transfusion and NEC progression, the overall quality of the evidence is low because of the lack of randomized controlled trials to determine causality.

## Introduction

Neonatal necrotizing enterocolitis (NEC) is a common and severe gastrointestinal emergency in neonatal intensive care units (NICUs), occurring most frequently in preterm infants born before 37 weeks of gestation and those with very low birth weight (VLBW), defined as a birth weight (BW) below 1500 g [1,2]. The pathogenesis of NEC is complex and involves multiple factors, including intestinal barrier dysfunction, an immature immune system, microbial dysbiosis, and local ischemia-reperfusion injury [2,3]. Despite significant advances in the diagnosis and management of NEC in recent years, severe NEC remains associated with high mortality, prolonged hospitalization, and potential for long-term sequelae such as short bowel syndrome [3], imposing a substantial burden on affected families.

Packed red blood cell (pRBC) transfusion, used to correct anemia or improve oxygen delivery, is a common therapeutic intervention in NICUs. However, pRBC transfusion can lead to various complications, including transfusion-associated NEC (TANEC). Evidence for an association between pRBC transfusion and NEC is derived mainly from observational studies, and whether pRBC transfusion is causally linked to NEC remains unclear [4]. Several previous studies have suggested that pRBC transfusion is also associated with disease severity in neonates [5–9], but current evidence is insufficient to establish a definitive association. No previous meta-analysis has systematically synthesized the available evidence to quantify this association.

This study aimed to systematically evaluate the potential association between pRBC transfusion, compared with non-transfusion management, and NEC progression by synthesizing the currently available evidence on the association between pRBC transfusion and NEC exacerbation. The findings may provide an evidence base for the clinical management of neonates with NEC and potentially improve outcomes.

## Materials and methods

### Study design

We conducted a systematic review and meta-analysis of the association between pRBC transfusion and NEC exacerbation. The study protocol was registered with PROSPERO (registration number: CRD420250655190), and the results were reported according to the Preferred Reporting Items for Systematic Reviews and Meta-Analyses (PRISMA) guidelines.

### Search strategy

We searched six English-language databases (Cochrane Library, EBSCO, Embase, Web of Science, Google Scholar, and PubMed) for studies on transfusion and NEC published up to May 10, 2025. We used the keywords (“necrotizing enterocolitis” OR “NEC”) AND (“transfusion” OR “blood transfusion”). Furthermore, the references of the included studies were also screened.

### Study selection

Two researchers reviewed the titles and abstracts and independently selected the studies. Subsequently, a comprehensive evaluation was conducted of the full texts of studies whose titles or abstracts met the inclusion criteria. Any disagreements were resolved by a third researcher. To be included, studies were required to meet all of the following criteria: case-control or cohort studies, or randomized controlled trials (RCTs), published in English, involving neonates with NEC, that compared pRBC transfusion with no transfusion and reported changes in NEC clinical status. Review articles, systematic reviews, case reports, editorials, animal studies, duplicate publications, and studies with incomplete data were excluded.

### Data extraction

Data extraction was performed independently by two researchers who extracted the following information: the first author’s name, year of publication, study location, study period, study design, gestational age (GA), BW, pRBC transfusion, number of NEC cases, outcomes of neonates with NEC, and transfusion protocols. Details of the variable extraction are provided in Supplementary Table 1. Any discrepancies between researchers were resolved through discussion between the two researchers or by consultation with a third researcher.

### Sensitivity analysis

We performed sensitivity analyses using two approaches: first, the leave-one-out method, and second, by excluding all studies that either reported statistically significant differences in GA and BW or did not report these parameters.

### Publication bias

We visually examined a funnel plot of the included studies to look for asymmetry as evidence of possible publication bias and evaluated the statistical significance of the asymmetry using Harbord’s modified test and Peter’s test. *p*-values < 0.05 were considered statistically significant.

### Quality evaluation

The quality of the observational studies was assessed using the Newcastle–Ottawa Scale (NOS) [10]. Two researchers scored the studies based on the quality of the selection of the study groups, the comparability of the groups, and the ability to assess the outcomes of interest. Studies were categorized as either high quality (9 points) or low quality (1–8 points). Any discrepancies between researchers regarding the quality of the studies were discussed and resolved by a third researcher.

### GRADE assessment

A “Summary of Findings” table was developed to assess the quality of the evidence using the Grading of Recommendations Assessment, Development, and Evaluation (GRADE) criteria. Observational studies were classified as providing low-certainty evidence. The quality of the evidence was downgraded if the study had limitations, inconsistencies, indirectness, or imprecision. Conversely, the quality of the evidence was upgraded if the study had a large magnitude of effect (i.e. when the relative risk was >2 or <0.5). The strength of the evidence was also be upgraded if the study demonstrated a dose-response gradient with a statistically significant test for trend.

### Statistical analysis

Statistical analyses were conducted using Stata 12.0 (StataCorp, College Station, TX, USA) and Review Manager version 5.3 (The Cochrane Collaboration, London, UK). The association between pRBC transfusion and worsening of NEC was expressed as odds ratios (ORs) with corresponding 95% confidence intervals (CIs). Statistical heterogeneity among studies was assessed using the Q statistic (with *p* < 0.1 considered significant) and the *I*^2^ statistic, with values of 25%, 50%, and 75% indicating low, moderate, and high heterogeneity, respectively. Owing to the anticipated heterogeneity among studies, a random-effects model using the inverse-variance method was applied in the meta-analysis.

## Results

### Study characteristics

**Figure 1.**
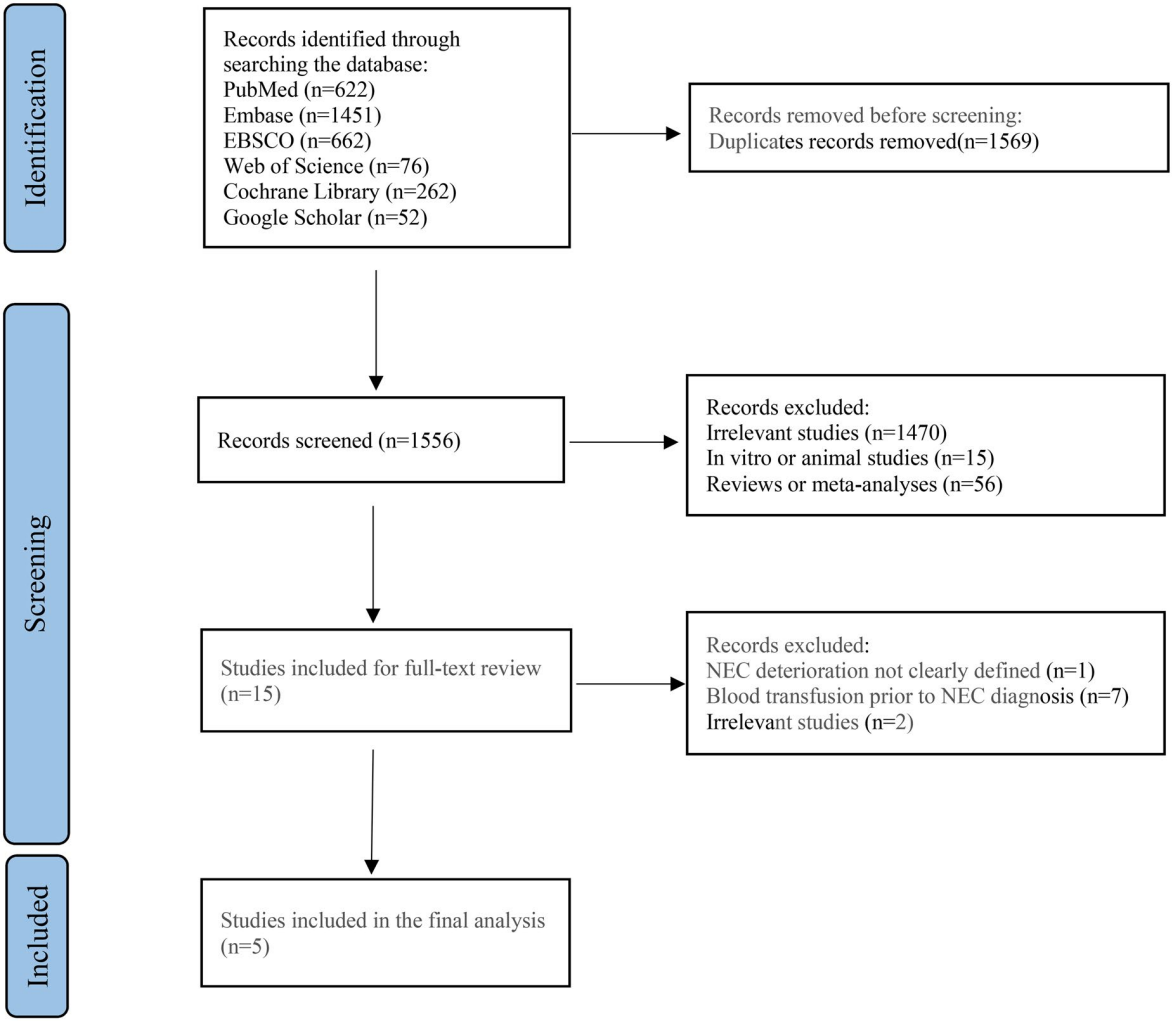
The flow diagram of studies selection.

We identified 3,125 articles published from the inception date of each database up to May 10, 2025, of which 1,569 were duplicate studies and were excluded. An additional 1,541 studies were excluded based on their titles and abstracts. We conducted a full-text evaluation of the remaining 15 studies. Of these studies, 10 were excluded because the pRBC transfusions occurred before the diagnosis of NEC or because the worsening of NEC was not clearly defined. The data from the remaining 5 studies, involving a total of 971 neonates, were pooled for the meta-analysis (Figure 1). All 5 studies were observational studies, including 3 case-control studies and 2 cohort studies. We did not identify any RCTs of the relationship between pRBC transfusion and NEC progression in neonates. All studies were published after 2014 (Table 1 and Supplementary Table 2), and two were rated as high quality (Table 2).

**Table 1.** The characteristics of the 5 studies included in the meta-analysis.

Study (author, year)	Country	Study design	Number	Patients	pRBC transfusion(n)	Outcomes(n)
Ibrohim et al., 2022 [6]	Indonesia	case-control study	214	premature neonates	Yes: 112 No: 102	deterioration: 33 16
Ahmed et al., 2015 [9]	USA	cohort Study	88	premature neonates	Yes: 51 No: 37	bowel resection: 50 17
Luo et al., 2015 [8]	China	case-control study	146	neonates	Yes: 31 No: 115	stage III NEC: 9 8
Garg et al., 2021 [7]	USA	case-control study	281	premature neonates	Yes: 164 No: 117	surgical non fulminant or fulminant NEC: 130 38
Luo et al., 2022 [5]	China	cohort study	242	near-term and full-term infants	Yes: 60 No: 182	stage III NEC: 23 17

NEC: necrotizing enterocolitis; pRBC: packed red blood cell.

**Table 2.** Quality assessment of the including case-control studies and cohort studies by the Newcastle-Ottawa scale.

Study (first author and year)	Study design	Selection	Comparability	Outcome	Total score
Ibrohim et al., 2022 [6]	case-control study	4	2	3	9
Ahmed et al., 2015 [9]	cohort study	4	2	3	9
Luo et al., 2015 [8]	case-control study	4	1	3	8
Garg et al., 2021 [7]	case-control study	4	0	3	7
Luo et al., 2022 [5]	cohort study	4	1	3	8

### pRBC transfusion and deterioration of NEC

**Figure 2.**
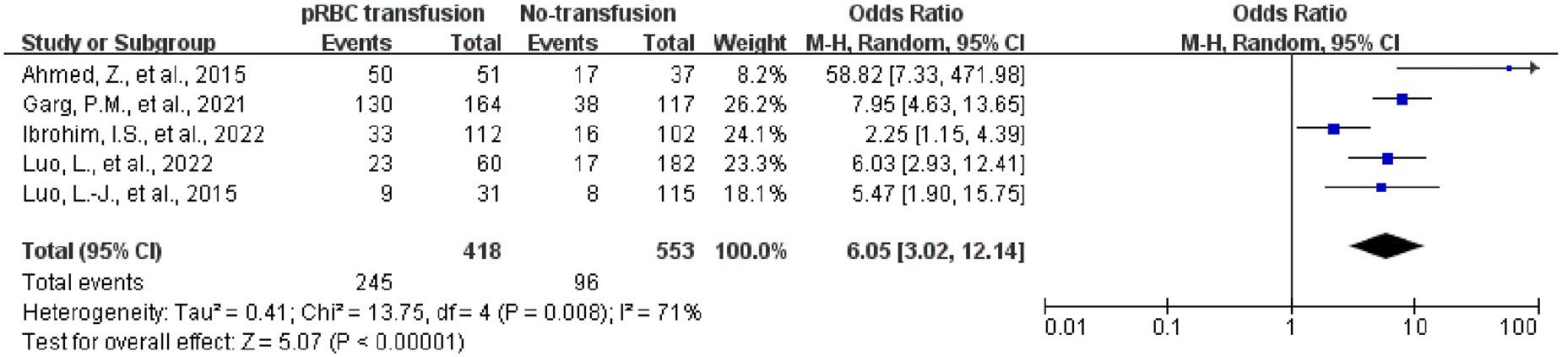
Forest plot of pRBC transfusion vs. no transfusion on NEC deterioration.

Because the *p*-value was <0.1 and the *I*^2^ value was >50%, indicating high heterogeneity, a random-effects model was used for the meta-analysis (Figure 2). The forest plot showed that pRBC transfusion was associated with an increased risk of NEC progression (OR: 6.05, 95% CI: 3.02–12.14) [5–9]. However, due to the limited number of included studies (*n* < 10), the results of the publication bias tests should be interpreted with caution, and the statistical power for subgroup analyses was limited.

### Sensitivity analyses

The association between pRBC transfusion and worsening NEC remained robust in a leave-one-out analysis with sequential removal of each individual study (Supplementary Table 3).

After excluding three studies with significant differences in GA or lacking GA data, analysis of the remaining two studies [5,9] showed that the association between pRBC transfusion and NEC deterioration remained significant (OR: 15.47, 95% CI: 1.53–156.08). Similarly, after excluding four studies with significant differences in BW between groups or lacking BW data, analysis of the remaining study [9] showed that the association between pRBC transfusion and NEC deterioration remained significant (OR: 58.82, 95% CI: 7.33–471.98). Due to the extremely small number of studies (two or fewer) and the resulting instability of these estimates, no robust conclusions can be drawn regarding whether GA or BW affected the overall results. These sensitivity analyses should be interpreted with extreme caution and are presented for exploratory purposes only.

### Publication bias

**Figure 3.**
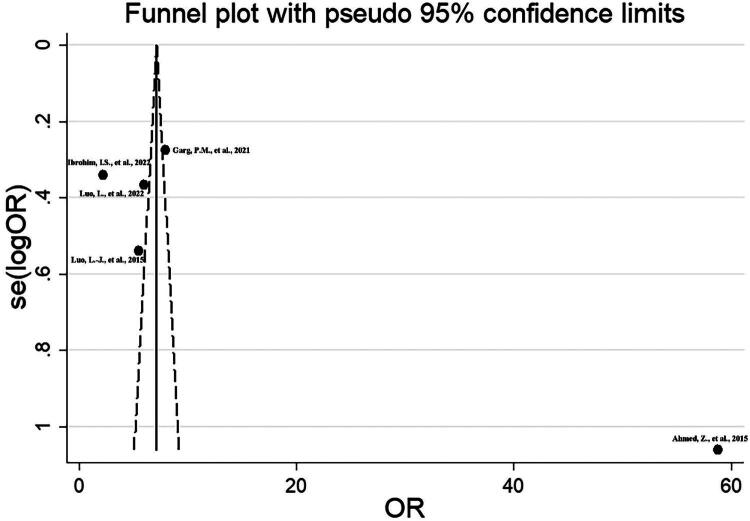
Funnel plot illustrating the impact of pRBC transfusion versus no transfusion on the deterioration of NEC.

The funnel plot is shown in Figure 3. Both Harbord’s modified test and Peters’ test showed no statistically significant evidence of publication bias (*p* = 0.564 and *p* = 0.228, respectively).

### GRADE assessment

In the GRADE assessment, the quality of evidence was downgraded because only observational studies were included, statistically significant differences in some baseline characteristics, such as GA and BW, were present among the included studies, and the heterogeneity analysis showed *p* < 0.1. However, the quality of evidence was upgraded because the relative risk was >2. In the final assessment, the quality of evidence was rated as “very low” (Table 3).

**Table 3.** The GRADE assessment of pRBC transfusion compared to no-transfusion for deterioration of neonatal NEC.

pRBC transfusion compared to no-transfusion for deterioration of neonatal NEC						
**Patient or population:** patients with deterioration of neonatal NEC **Settings:** neonatology **Intervention:** pRBC transfusion **Comparison:** no-transfusion						
Outcomes	Illustrative comparative risks* (95% CI)		Relative effect (95% CI)	No of Participants (studies)	Quality of the evidence (GRADE)	Comments
	Assumed risk	Corresponding risk				
	No-transfusion	Transfusion				
Deterioration of neonatal NEC Follow-up: mean 14 days	174 per 1000	566 per 1000 (390 to 728)	OR 6.21 (3.04 to 12.72)	971 (5 studies)	⊕⊝⊝⊝ very low^1,2,3,4^	
*The basis for the assumed risk (e.g. the median control group risk across studies) is provided in footnotes. The corresponding risk (and its 95% confidence interval) is based on the assumed risk in the comparison group and the relative effect of the intervention (and its 95% CI). CI: Confidence interval; OR: Odds ratio;						
GRADE Working Group grades of evidence High quality: Further research is very unlikely to change our confidence in the estimate of effect. Moderate quality: Further research is likely to have an important impact on our confidence in the estimate of effect and may change the estimate. Low quality: Further research is very likely to have an important impact on our confidence in the estimate of effect and is likely to change the estimate. Very low quality: We are very uncertain about the estimate.						
^1^Some studies showed statistically significant differences in important indicators such as gestational age or birth weight. ^2^Observational studies ^3^The heterogeneity analysis indicates that there is significant heterogeneity in this study. ^4^Relative risk >2						

NEC: necrotizing enterocolitis; pRBC: packed red blood cell.

## Discussion

In recent years, a growing body of evidence has indicated a potential association between pRBC transfusion and the development of NEC [4,11]. The effect of anemia combined with pRBC transfusion has been investigated using a neonatal rat model of NEC [12]. Some studies have shown a relationship between pRBC transfusion and disease severity in neonates with NEC [5–9]. However, to date, no systematic review has comprehensively assessed the strength of evidence for an association between pRBC transfusion and the subsequent development or worsening of NEC in neonates. This systematic review suggests an association between pRBC transfusion and subsequent deterioration of NEC. However, the limited number of available studies and the lack of randomized controlled trials (RCTs) may reduce statistical power, limit meaningful subgroup analyses, and render publication bias tests potentially unreliable. Therefore, these findings should be interpreted cautiously, and future studies are needed to confirm the results.

Through a synthesis of the available evidence, we found that deterioration of NEC in neonates may be associated with factors such as anemia, inflammatory injury, and microbial translocation [8,13,14].

A previous study found that, compared with infants with non-fulminant NEC, those with fulminant NEC received a higher number of pRBC transfusions within 48 h of NEC diagnosis [7]. Other studies have shown that patients with NEC have significantly lower preoperative hemoglobin levels and hematocrit values. The higher incidence of anemia among neonates with NEC may result from multiple contributing factors, including complex comorbidities and frequent blood sampling [5,9]. Anemia leads to decreased splanchnic oxygen saturation, resulting in tissue hypoxia and subsequent anaerobic metabolism, and may further aggravate intestinal injury by altering macrophage function, enhancing intestinal inflammatory responses, and compromising barrier integrity [13,15]. Such injury may trigger gut microbial translocation [16]. Notably, the severity of intestinal damage increases with increasing severity and duration of anemia [12]. In neonates with NEC, minimizing blood loss and conducting timely transfusion assessments are important, as pRBC transfusion therapy may potentially address intestinal hypoperfusion, hypoxia, and disrupted microbial diversity caused by anemia [17–19].

However, other studies have shown that pRBC transfusion has been associated with intestinal ischemia-reperfusion injury, which may be linked to exacerbation of NEC progression [20]. Therefore, in clinical situations in which pRBC transfusion is unavoidable for neonates with NEC, all feasible measures should be implemented to address potential risk factors for secondary intestinal injury in the context of transfusion. Levels of pro-inflammatory factors in blood products increase during storage. Transfusion of washed red blood cells can significantly reduce interleukin-6 (IL-6) production, thereby potentially attenuating inflammatory injury. This may mitigate potential factors associated with the exacerbation of NEC following transfusion [21]. Prolonged storage and storage-related reagents are associated with loss of red blood cell function and reduced nitric oxide levels [22]. These transfusion-related alterations have been associated with reduced oxygen delivery, followed by vasoconstriction and intestinal ischemic injury [22], which may also contribute to the exacerbation of NEC. Another study suggested that pRBC transfusion may be associated with inflammatory responses and immune dysregulation [23], representing another potential pathway that may correlate with NEC exacerbation. However, the underlying mechanisms require further investigation. Metabolic factors, in addition to blood compatibility and donor age, should be considered before neonatal transfusion. In addition, neonates with NEC should preferentially receive the freshest available units of pRBC or washed red blood cells.

This study identified a potential association between pRBC transfusion and the exacerbation of NEC progression; therefore, clinicians should carefully evaluate the risks and benefits of transfusion in NICU patients, particularly high-risk neonates. To mitigate the risk of NEC deterioration, proactive anemia prevention measures and optimized transfusion strategies are warranted. However, the evidence regarding whether the observed association reflects a causal relationship between pRBC transfusion and subsequent exacerbation of NEC is limited and of low quality because few studies have formally investigated this association, and all studies included in this review were observational. We did not identify any RCTs that addressed this question. Although our study did not establish a causal relationship between transfusion and NEC exacerbation, it provides direction for future research. Further multicenter, large-sample, well-designed studies are needed to clarify this relationship.

## Limitations

The review has several limitations. First, we searched only English-language databases, potentially missing studies published in other languages. Second, all studies included in the analysis were observational, and no RCTs were available; therefore, despite the observed association between pRBC transfusion and NEC deterioration, causality cannot be established. Third, owing to the limited number of included studies, statistical tests for publication bias and sensitivity analyses may be unreliable. The reported OR is highly susceptible to bias and may be inflated because of the observational design and lack of adjustment for key confounders. Some sensitivity analyses were based on only 1–2 studies, yielding unstable estimates. Thus, these effect sizes should be interpreted with caution. Furthermore, because of the limited available literature, we did not adopt a standardized definition of NEC deterioration. This heterogeneity may affect the validity of our findings, and future studies should use standardized definitions to enable meaningful meta-analyses. Fourth, confounding by indication and time-varying confounding or reverse causation cannot be ruled out. Infants with more severe anemia or advanced NEC are sicker and more likely to receive pRBC transfusions, and these same factors may independently increase the risk of NEC deterioration, potentially explaining the observed association. In addition, transfusions may be given after clinical deterioration begins, making it unclear whether the transfusion preceded or followed disease progression. This temporal ambiguity limits causal inference. Finally, because of the limited number of included studies, subgroup analyses could not be conducted according to the NEC severity, transfusion volume, number of transfusions, or timing of transfusion. These parameters may be associated with NEC progression and may provide valuable insights into the mechanisms underlying the observed association. However, due to insufficient available data, we were unable to incorporate these variables into the analysis. Future studies should systematically collect and report these variables to enable more comprehensive evaluations.

## Conclusion

Our study shows that pRBC transfusion may be associated with exacerbation of NEC in neonates. However, the optimal transfusion thresholds, timing, and volume for neonates with NEC require further evaluation. Although current evidence is insufficient to establish a causal relationship, this study provides insights for future research. Large-scale, well-designed studies are needed to further clarify the relationship between pRBC transfusion and NEC deterioration.

## Supplementary Material

Supplementary Table.docx

Supplementary checklist_PRISMA checklist.docx

## Data Availability

The data that support the findings of this study are available from the corresponding author, HW, upon reasonable request.

## References

[1] He Y, Zhang M, Tang J, et al. Mortality, morbidity, and care practices for 1750 very low birth weight infants, 2016-2021. Chin Med J (Engl). 2024;137(20):2452–2460. doi: 10.1097/CM9.0000000000002923. 38404117 PMC11479399

[2] Neu J, Walker WA. Necrotizing enterocolitis. N Engl J Med. 2011;364(3):255–264. doi: 10.1056/NEJMra1005408. 21247316 PMC3628622

[3] Duess JW, Sampah ME, Lopez CM, et al. Necrotizing enterocolitis, gut microbes, and sepsis. Gut Microbes. 2023;15(1):2221470. doi: 10.1080/19490976.2023.2221470. 37312412 PMC10269420

[4] Bellach L, Eigenschink M, Hassanein A, et al. Packed red blood cell transfusion in preterm infants. Lancet Haematol. 2022;9(8):e615–e626. doi: 10.1016/S2352-3026(22)00207-1. 35901846

[5] Luo L, Liu X, Yu H, et al. Red blood cell transfusions post diagnosis of necrotizing enterocolitis and the deterioration of necrotizing enterocolitis in full-term and near-term infants: a propensity score adjustment retrospective cohort study. BMC Pediatr. 2022;22(1):211. doi: 10.1186/s12887-022-03276-4. 35428277 PMC9012001

[6] Ibrohim IS, Pratama HA, Fauzi AR, et al. Association between prognostic factors and the clinical deterioration of preterm neonates with necrotizing enterocolitis. Sci Rep 2022; 12(1): 13911. doi: 10.1038/s41598-022-17846-0. 35978027 PMC9385610

[7] Garg PM, O’Connor A, Ansari MAY, et al. Hematological predictors of mortality in neonates with fulminant necrotizing enterocolitis. J Perinatol. 2021;41(5):1110–1121. doi: 10.1038/s41372-021-01044-3. 33772112 PMC7995678

[8] Luo L-J, Li X, Yang K-D, et al. Broad-spectrum antibiotic plus metronidazole may not prevent the deterioration of necrotizing enterocolitis from Stage II to III in full-term and near-term infants: a propensity score-matched cohort study. Medicine (Baltimore). 2015;94(42):e1862. doi: 10.1097/MD.0000000000001862. 26496340 PMC4620843

[9] Ahmed Z, Danielson L, Albeiruti R, et al. Blood transfusion in patients treated with surgery for necrotizing enterocolitis. Paediatr Anaesth. 2015;25(2):196–199. doi: 10.1111/pan.12485. 25041345

[10] Huang J, Zhang L, Tang J, et al. Human milk as a protective factor for bronchopulmonary dysplasia: a systematic review and meta-analysis. Arch Dis Child Fetal Neonatal Ed. 2019;104(2):F128–F136. doi: 10.1136/archdischild-2017-314205. 29907614

[11] Su Y, Xu R-H, Guo L-Y, et al. Risk factors for necrotizing enterocolitis in neonates: a meta-analysis. Front Pediatr. 2022;10:1079894. doi: 10.3389/fped.2022.1079894. 36683790 PMC9853297

[12] MohanKumar K, Namachivayam K, Song T, et al. A murine neonatal model of necrotizing enterocolitis caused by anemia and red blood cell transfusions. Nat Commun. 2019;10(1):3494. doi: 10.1038/s41467-019-11199-5. 31375667 PMC6677753

[13] Maheshwari A. Severe anemia predisposes very premature infants to transfusion-associated necrotizing enterocolitis. Semin Fetal Neonatal Med. 2025;30(1):101615. doi: 10.1016/j.siny.2025.101615. 40059009

[14] Susan DR, Naomi LCL, Catherine SM. Guidelines for assessing appropriateness of pediatric transfusion. Transfusion. 2002;42(11):1398–1413. doi: 10.1046/j.1537-2995.2002.00208.x. 12421212

[15] Arthur CM, Nalbant D, Feldman HA, et al. Anemia induces gut inflammation and injury in an animal model of preterm infants. Transfusion. 2019;59(4):1233–1245. doi: 10.1111/trf.15254. 30897226 PMC6525338

[16] Pammi M, Hollister E, Neu J. Gut injury and the microbiome in neonates. Clin Perinatol. 2020;47(2):369–382. doi: 10.1016/j.clp.2020.02.010. 32439117

[17] Banerjee J, Leung TS, Aladangady N. Blood transfusion in preterm infants improves intestinal tissue oxygenation without alteration in blood flow. Vox Sang. 2016;111(4):399–408. doi: 10.1111/vox.12436. 27509230

[18] Yracheta J, Muraoka W, Wu X, et al. Whole blood resuscitation restores intestinal perfusion and influences gut microbiome diversity. J Trauma Acute Care Surg. 2021;91(6):1002–1009. doi: 10.1097/TA.0000000000003381. 34407003

[19] Zheng SC, He S. The near-infrared spectroscopy to evaluate neonatal improvement after transfusion: a systematic review and meta-analysis. BMC Pediatr. 2025;25(1):385. doi: 10.1186/s12887-025-05731-4. 40375204 PMC12080153

[20] Iijima S. Clinical dilemma involving treatments for very low-birth-weight infants and the potential risk of necrotizing enterocolitis: a narrative literature review. J Clin Med. 2023;13(1):62. doi: 10.3390/jcm13010062. 38202069 PMC10780023

[21] Cholette JM, Henrichs KF, Alfieris GM, et al. Washing red blood cells and platelets transfused in cardiac surgery reduces postoperative inflammation and number of transfusions. Pediatr Crit Care Med. 2012;13(3):290–299. doi: 10.1097/PCC.0b013e31822f173c. 21926663 PMC3839819

[22] James DR, Gregory SA, Michael A, et al. S-nitrosohemoglobin deficiency: a mechanism for loss of physiological activity in banked blood. Proc Natl Acad Sci U S A. 2007;104(43):17058–17062. doi: 10.1073/pnas.0707958104. 17940022 PMC2040473

[23] D’Alessandro A, Zimring JC. From metabolomics to transfusion-associated immunomodulation. Curr Opin Immunol. 2025;96:102646. doi: 10.1016/j.coi.2025.102646. 40848579 PMC13407375

