# Supplementary material for: Association between packed red blood cell transfusion and clinical deterioration in neonatal necrotizing enterocolitis: a systematic review and meta-analysis
Source: Ann Med. 2026 May 24;58(1):2675123. doi: 10.1080/07853890.2026.2675123 (PMC13202688; doi:10.1080/07853890.2026.2675123)
Supplement: Supplementary Table.docx [file IANN_A_2675123_SM2367.docx]

Supplementary table 1 The detailed variable extraction table.

| Category | Variable Name Extracted | Description | Type | Handling in Analysis |
| --- | --- | --- | --- | --- |
| Study Identification | Study ID | First author & publication year | N/A | Unique identifier for referencing studies in tables, figures, and throughout the analysis. Not used in statistical models. |
| Study Identification | Study design | RCT, Cohort, Case-control | N/A | To define the type and evidence grade |
| Study Characteristics | Country | Country where the study was conducted | Categorical (as reported) | To explore geographical heterogeneity; To assess potential sources of clinical or methodological heterogeneity and to inform considerations for subgroup analyses. |
| Participant Characteristics | Total sample size | Count (n) | N/A | For weighting in meta-analysis |
| Participant Characteristics | Sample size per group | Count (n) for Exposure /Control groups | N/A | For calculating effect sizes |
| Participant Characteristics | Gestational age category | premature neonates (<37 weeks) / Term (≥37 weeks)/ neonates | Categorical (as reported, uncorrected) | To characterize the study population and assess baseline comparability between groups as reported in the original studies. |
| Participant Characteristics | Gestational age | Weeks（median or mean±SD） | mean±SD OR median | To explore geographical heterogeneity; To assess potential sources of clinical or methodological heterogeneity and to inform considerations for subgroup analyses. |
| Participant Characteristics | Birth weight | grams (mean±SD) | mean±SD | To explore geographical heterogeneity; To assess potential sources of clinical or methodological heterogeneity and to inform considerations for subgroup analyses. |
| Exposure | Transfusion protocols | A defined set of criteria (e.g., Hb/Hct level, clinical symptoms) guiding the decision to administer packed red blood cells | Categorical (as reported, uncorrected) | Will be used primarily to investigate sources of heterogeneity in the meta-analysis, as well as to explore details for optimizing transfusion management, such as transfusion volume and threshold criteria. |
| Exposure | pRBC transfusion | Count (n) for Exposure/Control groups | Categorical (as reported, uncorrected) | Will be used as the primary exposure variable in the meta-analysis |
| Primary Outcomes | NEC deterioration | Count (n) for Exposure/Control groups | Categorical (as reported, uncorrected) | Will be used as the primary Outcomes variable in the meta-analysis |

Supplementary table 2 GA, BW and transfusion protocols of the 5 included studies

| Study (author, year) | GA (weeks) | BW (grams) | Transfusion protocols |
| --- | --- | --- | --- |
| Ibrohim, I.S., et al., 2022 | Not reported | Not reported | Not reported |
| Ahmed, Z., et al., 2015 | (mean±SD)  30 ± 4  29 ± 4 | (mean±SD)  1200± 80  1300±70 | Not reported |
| Luo, L.-J., et al., 2015 | Not reported | Not reported | Not reported |
| Garg, P.M., et al., 2021 | Not reported | Not reported | pRBC transfusions are considered if the hematocrit is ≤30% (hemoglobin ≤ 10 gm/dL) for neonates requiring moderate or significant mechanical (invasive/non-invasive) ventilation, defined as mean airway pressure >8cm H_2_O and FiO2 > 0.40 on a conventional ventilator. |
| Luo, L., et al., 2022 | (median)  38.1 (36.1–39.5)  38.9 (37.3–39.7) | (mean±SD)  2763±614  2989±580 | pRBC transfusion was administered according to clinical need within guideline recommendations, at a dose of 10~20 ml/kg over 2~4 hours, without routine fasting. |

GA: gestational age; BW: birth weight; pRBC: packed red blood cell.

Supplementary table 3 The results of sensitivity analyses.

| The leave-one-out method |  | Odds ratios | 95% confidence intervals |
| --- | --- | --- | --- |
| Included studies | Exclude studies |  |  |
| Ahmed, Z., et al., 2015  Luo, L.-J., et al., 2015  Garg, P.M., et al., 2021  Luo, L., et al., 2022  Included studies | Ibrohim, I.S., et al., 2022  Exclude studies | 7.68 | 4.43-13.31 |
| Ibrohim, I.S., et al., 2022  Luo, L.-J., et al., 2015  Garg, P.M., et al., 2021  Luo, L., et al., 2022  Included studies | Ahmed, Z., et al., 2015  Exclude studies | 4.95 | 2.70-9.09 |
| Ahmed, Z., et al., 2015  Ibrohim, I.S., et al., 2022  Garg, P.M., et al., 2021  Luo, L., et al., 2022  Included studies | Luo, L.-J., et al., 2015  Exclude studies | 6.40 | 2.71-15.09 |
| Ahmed, Z., et al., 2015  Ibrohim, I.S., et al., 2022  Luo, L.-J., et al., 2015  Luo, L., et al., 2022  Included studies | Garg, P.M., et al., 2021  Exclude studies | 5.79 | 2.30-14.59 |
| Ahmed, Z., et al., 2015  Ibrohim, I.S., et al., 2022  Luo, L.-J., et al., 2015  Garg, P.M., et al., 2021 | Luo, L., et al., 2022 | 6.44 | 2.48-16.70 |
| Included studies with no statistically significant difference in gestational age | Exclude studies with significant differences in, or without data on, gestational age |  |  |
| Ahmed, Z., et al., 2015  Luo, L., et al., 2022 | Ibrohim, I.S., et al., 2022  Luo, L.-J., et al., 2015  Garg, P.M., et al., 2021 | 15.47 | 1.53-156.08 |
| Included studies with no statistically significant difference in birth weight | Exclude studies with significant differences in, or without data on, birth weight |  |  |
| Ahmed, Z., et al., 2015 | Ibrohim, I.S., et al., 2022  Luo, L.-J., et al., 2015  Garg, P.M., et al., 2021  Luo, L., et al., 2022 | 58.82 | 7.33-471.98 |
